# Sex-specific phenotypes of hyperthyroidism and hypothyroidism in mice

**DOI:** 10.1186/s13293-016-0089-3

**Published:** 2016-08-24

**Authors:** Helena Rakov, Kathrin Engels, Georg Sebastian Hönes, Karl-Heinz Strucksberg, Lars Christian Moeller, Josef Köhrle, Denise Zwanziger, Dagmar Führer

**Affiliations:** 1 Division of Laboratory Research Department of Endocrinology and Metabolism, Clinical Chemistry, University Hospital Essen, University Duisburg-Essen, 45122 Essen, Germany; 2 Charité-Universitätsmedizin Berlin, Institute of Experimental Endocrinology, 13353 Berlin, Germany

**Keywords:** Thyroid hormone transport, Thyroid hormone action, Hyperthyroidism, Hypothyroidism, Sex difference, Sex steroid hormone, Mice

## Abstract

**Background:**

Thyroid dysfunction is more common in the female population, however, the impact of sex on disease characteristics has rarely been addressed. Using a murine model, we asked whether sex has an influence on phenotypes, thyroid hormone status, and thyroid hormone tissue response in hyper- and hypothyroidism.

**Methods:**

Hypo- and hyperthyroidism were induced in 5-month-old female and male wildtype C57BL/6N mice, by LoI/MMI/ClO_4_^−^ or T_4_ i.p. treatment over 7 weeks, and control animals underwent sham treatment (*N* = 8 animals/sex/treatment). Animals were investigated for impact of sex on body weight, food and water intake, body temperature, heart rate, behaviour (locomotor activity, motor coordination, and strength), liver function, serum thyroid hormone status, and cellular TH effects on gene expression in brown adipose tissue, heart, and liver.

**Results:**

Male and female mice showed significant differences in behavioural, functional, metabolic, biochemical, and molecular traits of hyper- and hypothyroidism. Hyperthyroidism resulted in increased locomotor activity in female mice but decreased muscle strength and motor coordination preferably in male animals. Hypothyroidism led to increased water intake in male but not female mice and significantly higher serum cholesterol in male mice. Natural sex differences in body temperature, body weight gain, food and water intake were preserved under hyperthyroid conditions. In contrast, natural sex differences in heart rate disappeared with TH excess and deprivation. The variations of hyper- or hypothyroid traits of male and female mice were not explained by classical T_3_/T_4_ serum state. TH serum concentrations were significantly increased in female mice under hyperthyroidism, but no sex differences were found under eu- or hypothyroid conditions. Interestingly, analysis of expression of TH target genes and TH transporters revealed little sex dependency in heart, while sex differences in target genes were present in liver and brown adipose tissue in line with altered functional and metabolic traits of hyper- and hypothyroidism.

**Conclusions:**

These data demonstrate that the phenotypes of hypo- and hyperthyroidism differ between male and female mice and indicate that sex is an important modifier of phenotypic manifestations.

**Electronic supplementary material:**

The online version of this article (doi:10.1186/s13293-016-0089-3) contains supplementary material, which is available to authorized users.

## Background

Thyroid dysfunction, i.e., hyper- or hypothyroidism, occurs with 2- to 9-fold higher prevalence in women [1], yet besides fertility aspects and bone metabolism, a possible impact of gender on disease characteristics has not been well studied, neither in the clinical setting nor in epidemiological cohorts.

Experimental approaches using murine animal models to study thyroid hormone (TH) action so far mostly include mice of only one sex or, occasionally, do not even specify the mouse sex. A momentum calling for increased awareness of sex impact on manifestation, prognosis, and treatment of diseases was published in Nature in 2014 and was afterwards re-emphasized by the Endocrine Society [2, 3]. Furthermore, the “Guide to investigating thyroid hormone economy and action in rodent and cell models analysis of TH action” published by the American Thyroid Association in 2013 advised to study male and female rodents separately as the response to TH may be sexually dimorphic [4].

In view of the clinical situation where both women and men are affected by hyper- and hypothyroidism and consequences of disease may be gender-specific, we decided to employ a murine animal model, in which TH function status can be easily manipulated, for a comprehensive characterization of sex impact on TH action. Hence, male and female mice were studied for changes in behavioural, functional, metabolic, and biochemical markers in addition to the analysis of cellular TH effects on gene expression in selected target organs comprising brown adipose tissue (BAT), heart, and liver under conditions of TH excess and deprivation.

## Methods

### Animals and study design

Male and female C57BL/6NTac (*N* = 8/sex/treatment; Taconic Europe A/S, Denmark) mice aged 5 months were housed in temperature- (23 ± 1 °C) and light-controlled (inverse 12:12 h light-dark cycle) conditions. Food and water were provided ad libitum. All animal experiments were performed in accordance with the German regulations for Laboratory Animal Science (GVSOLAS) and the European Health Law of the Federation of Laboratory Animal Science Associations (FELASA). The protocols for animal studies were approved by the *Landesamt für Natur, Umwelt und Verbraucherschutz Nordrhein-Westfalen* (LANUV-NRW). All efforts were made to minimize suffering.

**Fig. 1:**
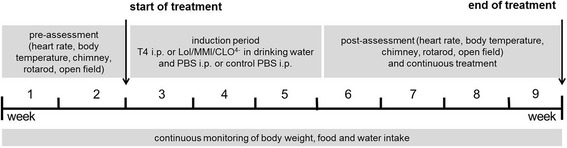
Study design for phenotypic characterization of hyper- and hypothyroidism in female and male mice. Two weeks were used to study the euthyroid control state of male and female mice (run-in period), followed by 3 weeks induction period without experiments other than monitoring body weight and food and water intake. After the induction period, male and female mice were analyzed under hyper- and hypothyroid conditions over a period of 4 weeks and compared to sham-treated controls

The 9-week experimental period was divided into three parts (Fig. 1), consisting of a 2-week run-in period prior to the manipulation of thyroid status to phenotypically characterize each individual mouse (pre-assessment), a 3-week treatment period to induce hyper- or hypothyroidism, and a 4-week assessment period to repeat the phenotypic characterization of each individual animal under chronic TH manipulation or euthyroid control treatment.

Chronic hyperthyroidism and hypothyroidism were induced as previously described [5, 6]. Briefly, for hyperthyroidism, i.p. injections of 1 μg/g body weight T_4_ (Sigma-Aldrich (T2376), USA) were performed every 48 h. For the induction of chronic hypothyroidism, animals were fed a low-iodine diet (LoI; TD.95007, Harlan Laboratories, USA) and received drinking water supplemented with 0.02 % methimazole (MMI, Sigma-Aldrich (301507), USA), 0.5 % sodium perchlorate (ClO_4_^−^) (Sigma-Aldrich (310514), USA), and 0.3 % saccharine as sweetener (Sigma-Aldrich (240931), USA) (LoI/MMI/ClO_4_^−^). In addition, hypothyroid animals received i.p. injections of PBS every 48 h. Control animals were fed a control diet and received i.p. injections of PBS every 48 h.

### Blood sample collection, serum TH, triglycerideand cholesterol measurements

Final blood samples were stored 30 min on ice and centrifuged, and free triiodothyronine (fT_3_), free thyroxine (fT_4_), and total T4 (TT_4_) concentrations in serum of mice were measured using commercial ELISA kits according to the manufacturer’s instructions (DRG Instruments GmbH, Marburg, Germany). Detection limits were 0.5 μg/dL, 0.05 ng/dL, and 0.05 pg/mL for TT_4_, fT_4_, and fT_3_, respectively. Serum TSH was measured with a sensitive, heterologous, disequilibrium double-antibody precipitation radioimmunoassay with a detection limit of 10 mU/L [7] (kindly performed by the laboratory of Prof. Refetoff at the University of Chicago, Chicago, USA). Total cholesterol concentrations in serum were detected using an enzymatic cholesterol quantitation kit (Sigma-Aldrich (MAK043), USA) according to the manufacturer’s instructions. Total triglyceride concentrations in serum were detected using an enzymatic serum triglyceride determination kit (Sigma-Aldrich (TR0100), USA) according to the manufacturer’s instructions.

### Monitoring of body weight, food, and water intake

Body weight was measured two to three times a week by placing mice on a scale. Food consumption was determined once a week by measuring the weight of remaining food pellets in the metal cage top. Water intake was controlled by weighting of water bottles (for all to the nearest 0.1 g) weekly or twice per week in control and TH-manipulated groups. Male mice were caged individually, while female mice were kept in groups of three to four animals. Thereby, food and water intake of female mice was calculated by dividing the measured intake by the number of animals in each cage.

For each mouse, average daily food and water intake was calculated, and adjustments for body weight were derived by dividing the average intake by the average body weight and multiplying the result by 40 g.

### Measurement of body temperature

Body temperature of mice was assessed four times using a cream-covered rectal probe (RET-3 rectal probe for mice, Kent Scientific Corporation, USA) connected to a thermocouple thermometer (Acorn Temp JKT Thermocouple Meter, Kent Scientific Corporation, USA). Mice were placed on the top of the cage, and the rectal probe was carefully inserted 2 cm into the rectum until a steady temperature was measured, which took approximately 8 to 10 s.

### Measurement of heart rate

Non-invasive restrained ECG recording was performed using an in-house protocol [8]. Conscious mice were placed on a platform with their paws on silver electrodes and were restrained by a half-tunnel. Signal was derived, enhanced, and digitalized (Picoscope 2204, Pico Technology, UK). ECG was recorded using Picoscope 6 Software over 60–90 s, and heart rate was determined by measurements of RR intervals over 8 s in a stable steady state. Non-invasive restrained ECG was recorded three times in all animals.

### Collection of organs

As previously described [5, 6], animals were euthanized 24 h after the last T_4_ treatment or continuous TH deprivation. Liver, heart, and BAT were isolated and stored at −80 °C until further processing.

### Isolation of RNA, cDNA synthesis, and real-time PCR

RNA extraction was performed as previously described [5, 6]. Briefly, total RNA was extracted using RNeasy mini kit (Qiagen, Germany) and reversed transcribed using SuperScript III First-Strand Synthesis System for RT-PCR according to instruction manuals (Life Technologies, Germany). Exon spanning primers for amplification of TH-responsive genes (Additional file 1: Table S1) were designed using PrimerBlast (NCBI) and synthesized by Eurofins (Eurofins MWG Synthesis, Germany). Quantitative real-time PCR (qRT-PCR) was performed using LightCycler® DNA Master SYBR Green I and the LightCycler®480 System (Roche, Germany). The PCR program consisted of an initial denaturation step (5 min at 95 °C) and 40 amplification cycles with 15 s at 95 °C, 10 s at 60 °C, and 20 s at 72 °C.

For the normalization of gene expression, reference genes *18S*, *Ppia* (peptidylprolyl isomerase A, cyclophilin A), and *Rpl13 a* (ribosomal protein L13a) for liver; *18S*, *Gapdh* (glyceraldehyde-3-phosphate dehydrogenase), and *Polr2a* (polymerase RNA II) for heart; and *18S*, *Ppia*, and *Gapdh* for BAT were used. The following genes were studied as TH responsive: *Dio1* (deiodinase 1), *Dio2* (deiodinase 2), *Tbg* (thyroxine-binding globulin), *Me1* (malic enzyme 1), *Myh6* (myosin heavy chain 6), *Hcn4* (hyperpolarization activated cyclic nucleotide gated potassium channel 4), *Ucp1* (uncoupling protein 1), and *Pgc1α* (peroxisome proliferator-activated receptor gamma coactivator 1-alpha) [4, 9–11]. To calculate the relative quantification ratio, an efficiency-corrected calculation model was used [12–14].

### Behavioural tests

#### Rotarod test

The rotarod test [15] basically consists of five 3-cm diameter cylinders, enabling five mice to be tested simultaneously. On the first testing day, mice were allowed to acclimate to the rotarod test by letting them walk 6 min on the rotating cylinder with constant acceleration from 2–20 rpm.

For each rotarod session, mice were subjected to four trials, with a minimum resting time interval of 6 min between the trials. Rotation mode was switched to constant acceleration from 4–50 rpm within 5 min. Maximum time and speed mastered by the animal were recorded. Mice that fell off the rod or attained full speed were placed back to their home cages. Every animal was subjected to two rotarod sessions (with a suspension period of 7 days) each before and after the induction of thyroid dysfunction (four sessions with 16 trials in total). Sessions 2 and 4 were used for statistical analysis.

#### Chimney test

The chimney test is constituted of a plastic tube (length 30 cm, diameter 3 cm). Mice were placed inside the tube and allowed to reach the other end. Then, the tube was turned into a vertical position with mice head upside down. The test consisted of determining the time taken by mice to climb up to 25 cm of height. Mice were given 90 s of time to pass the test [16, 17].

### Open Field

Open field consisted of a closed square area made of Plexiglas (50 × 50 cm). The area was divided into four corners, four walls, and a center region (16 × 16 cm). Animals were tested in the dark phase of their dark/light cycle. Mice were placed in the center of the open field and allowed to move freely for 5 min. Movements were monitored and digitalized by VideoMot2 software. Software recorded entries in all areas including time, frequency, latency, and distance. Occurring events of rearing, freezing, grooming, and jumping were recorded manually by the investigator during the experiment. Mice were placed in their home cages after 5 min of exploring the area.

### Statistical analysis

All data are shown as means ± standard deviation (SD) or standard error of the mean (SEM), as indicated. Statistical analysis using GraphPad Prism 6 Software was performed. Two-way ANOVA was used to compare more than two groups, followed by the Bonferroni post hoc analysis. The effects of both thyroid dysfunctions are often opposing, and inclusion of both treatments would therefore always show a significant treatment effect. To prevent false positive results, statistical analyses of treatment groups were performed separately for hyper- and hypothyroid groups. Unpaired Student’s *t* test to compare differences between two groups was applied. Values of *p < 0.05, **p < 0.01, and ***p < 0.001 were considered statistically significant.

## Results

### Sex modifies the impact of TH on body weight, food and water consumption, body temperature, and heart rate in hyper- and hypothyroid state

**Fig. 2:**
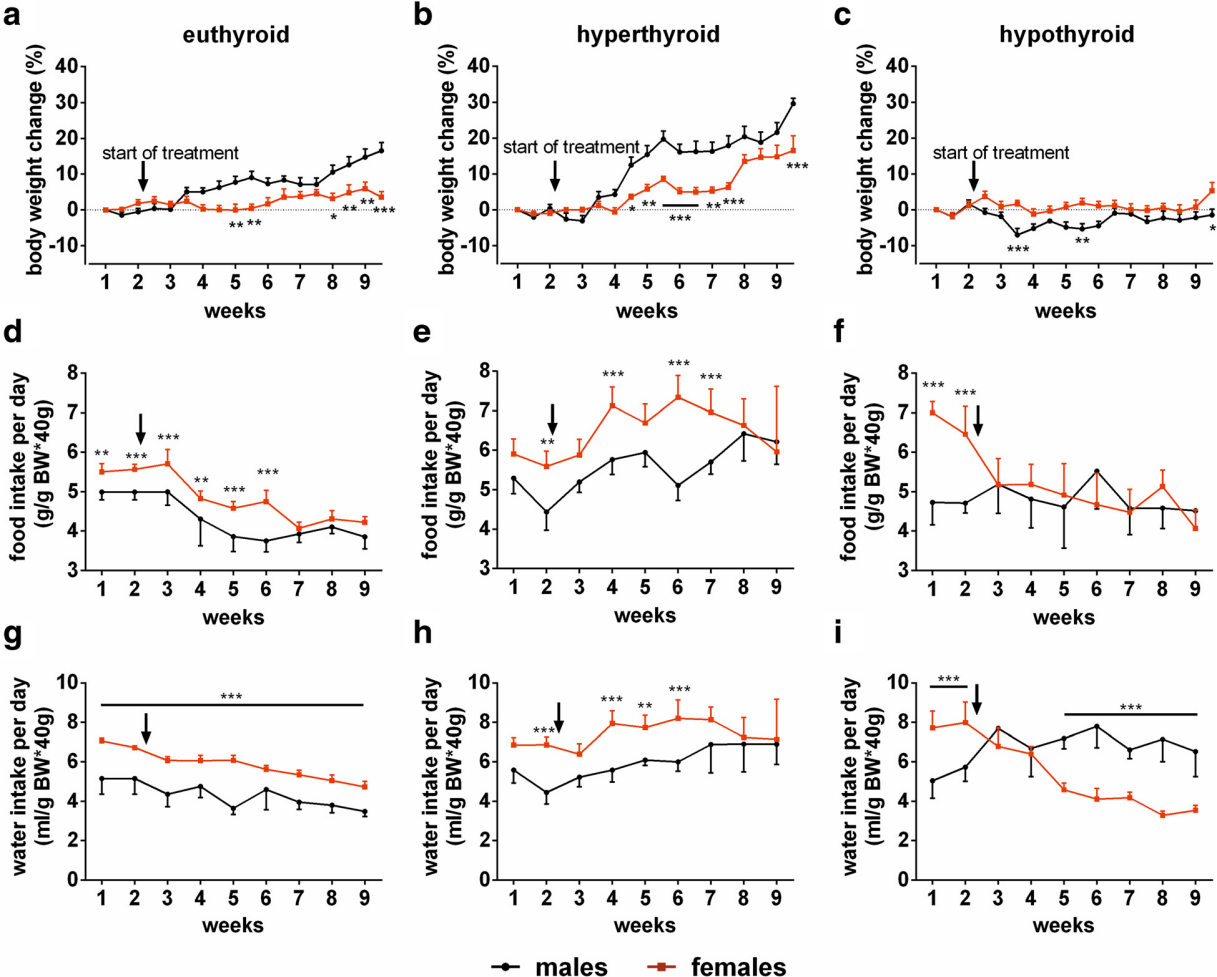
Body weight change, food and water intake in euthyroid, T_4_ or LoI/MMI/ClO_4_^−^ treated mice. Time course of the average body weight (BW) of male and female mice over an experimental period of 9 weeks under **a** control, **b** T_4_, and **c** LoI/MMI/ClO_4_^−^ treatment. Average food intake was related to BW during experiment in **d** euthyroid, **e** hyperthyroid, and **f** hypothyroid conditions in mice of both sexes. After run-in period mice were placed on low-iodine diet for the induction of hypothyroidism, or on control iodine diet by the same supplier to adapt the nutritional intake (euthyroid and hyperthyroid groups). *Arrows* indicate the start of treatment. Time course of average water intake was monitored over the experimental period of 9 weeks under **g** control, **h** TH excess, and **i** TH deprivation of male and female mice. Data are presented as mean ± SD, *N* = 8 animals/sex/treatment/time point; two-way ANOVA followed by the Bonferroni post hoc analysis applied for time and sex effects, *p < 0.05, **p < 0.01, ***p < 0.001 above *graph* represent multiple-testing results

Male mice showed significantly increased body weight (BW) compared to female mice under euthyroid and hyperthyroid conditions (Fig. 2a, b). For euthyroid mice, the highest ΔBW was observed in week 9, *m* < 16.5 % and *f* <5.9 % (*F*_(17,252)_ = 9.003, *p* < 0.0001 for time effect and *F*_(1,252)_ = 67.18, *p* < 0.0001 for sex effect, interaction: *F*_(17,252)_ = 4.065, *p* < 0.0001). Similarly, for hyperthyroid mice, the highest ΔBW was observed in week 9, *m* < 29.6 % vs *f* < 16.5 % (*F*_(17,252)_ = 33.43, *p* < 0.0001 for time effect and *F*_(1,252)_ = 88.28, *p* < 0.0001 for sex effect, interaction *F*_(17,252)_ = 3.966, *p* < 0.0001). In contrast, sex difference in body weight gain disappeared in hypothyroidism except for occasional time points in weeks 3, 5, and 9 (highest ΔBW *m* ~2 % at week 9 vs *f* ~0.7 % at week 9, *F*_(17,252)_ = 2.055, *p* = 0.0093 for time effect and *F*_(1,252)_ = 52.98, *p* < 0.0001 for sex effect with no interaction *F*_(17,252)_ = 1.614, *p* = 0.0605, Fig. 2c).

Euthyroid female mice consumed more food (*m* ~4.5 vs *f* ~5 g/g BW*40 g) and water (*m* ~4.8 vs *f* ~6 ml/g BW*40 g) than male mice (Fig. 2d, g; food intake: *F*_(8,126)_ = 61.77, *p* < 0.0001 for time effect and *F*_(1,126)_ = 121.7, *p* < 0.0001 for sex effect, interaction: *F*_(8,126)_ = 3.469, *p* = 0.0012; water intake: *F*_(8,126)_ = 31.92, *p* < 0.0001 for time effect and *F*_(1,126)_ = 385.3, *p* < 0.0001 for sex effect, interaction: *F*_(8,126)_ = 3.342, *p* = 0.0017). T_4_ administration enhanced food (*m* ~5.5 vs *f* ~6.5 g/g BW*40 g) and water intake (*m* ~6 vs *f* ~7.5 ml/g BW*40 g) in both sexes, again significantly more pronounced in female mice (Fig. 2e, h; food intake: *F*_(8,126)_ = 11.56, *p* < 0.0001 for time effect and *F*_(1,126)_ = 78.90, *p* < 0.0001 for sex effect, interaction: *F*_(8,126)_ = 5.721, *p* < 0.0001, water intake: *F*_(8,126)_ = 7.898, *p* < 0.0001 for time effect and *F*_(1,126)_ = 90.29, *p* < 0.0001 for sex effect, interaction: *F*_(8,126)_ = 3.170, *p* = 0.0026). Hypothyroidism abolished sex difference in food intake (*m* ~5 vs *f* ~4.8 g/g BW*40 g, *F*_(8,126)_ = 9.004, *p* < 0.0001 for time effect and *F*_(1,126)_ = 15.25, *p* = 0.0002 for sex effect, interaction: *F*_(8,126)_ = 9.393, *p* < 0.0001, Fig. 2f) and reversed sex difference in water consumption with female mice showing significantly less water intake (*m* ~6.8 vs *f* ~4 ml/g BW*40 g, *F*_(8,126)_ = 13.55, *p* < 0.0001 for time effect and *F*_(1,126)_ = 90.96, *p* < 0.0001 for sex effect, interaction: *F*_(8,126)_ = 34.92, *p* < 0.0001, Fig. 2i).

**Fig. 3:**
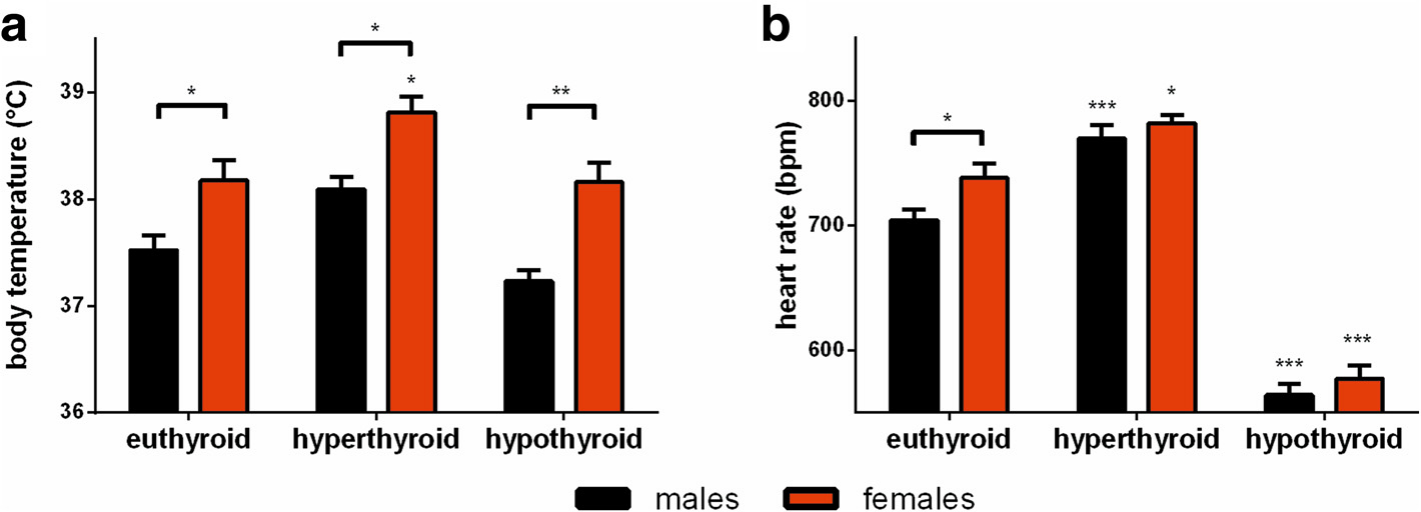
Influence of sex and change of TH serum concentrations on body temperature and heart rate. **a** Body temperature was assessed by rectal temperature measurements and **b** non-invasive ECG was performed on conscious mice of both sexes under euthyroid, hyperthyroid, and hypothyroid conditions. Data are presented as mean ± SD, *N* = 8 animals/sex/treatment; two-way ANOVA followed by the Bonferroni post hoc analysis applied for treatment and sex effects, *p < 0.05, **p < 0.01, ***p < 0.001 above *bars* represent multiple-testing results

Body temperature, measured by a rectal probe, was higher in euthyroid female compared to male mice (*m* ~37.5 vs *f* ~38.2 °C, *p* < 0.05). This sex difference persisted during T_4_ administration (*m* ~38.1 vs *f* ~38.8 °C, *p* < 0.05, *F*_(1,28)_ = 21.23, *p* < 0.0001 for sex effect, *F*_(1,28)_ = 16.50, *p* = 0.0004 for treatment effect and *F*_(1,28)_ = 0.04857, *p* = 0.8272 for interaction) and LoI/MMI/ClO_4_^−^ treatment (*m* ~37.2 vs *f* ~38.2 °C, *p* < 0.01, *F*_(1,28)_ = 25.77, *p* < 0.0001 for sex effect, *F*_(1,28)_ = 0.9667, *p* = 0.3339 for treatment effect, and *F*_(1,28)_ = 0.7794, *p* = 0.3848 for interaction, Fig. 3a). Interestingly, a drop in body temperature was only observed in male but not female hypothyroid mice compared to euthyroid controls.

Non-invasive ECG measurements were performed to investigate the influence of TH on heart rate (HR). Euthyroid female animals showed higher HR than male mice (*m* ~704 vs *f* ~738 bpm, *p* < 0.05). Sex difference in HR disappeared with TH excess (*m* ~770 vs *f* ~782 bpm, *F*_(1,28)_ = 5.837, *p* = 0.0225 for sex effect, *F*_(1,28)_ = 32.65, *p* < 0.0001 for treatment effect, and *F*_(1,28)_ = 1.306, *p* = 0.2628 for interaction) or deprivation (*m* ~564 vs *f* ~577 bpm, *F*_(1,28)_ = 5.586, *p* = 0.0253 for sex effect, *F*_(1,28)_ = 227.2, *p* < 0.0001 for treatment effect, and *F*_(1,28)_ = 1.099, *p* = 0.3034 for interaction, Fig. 3b).

### Male mice show pronounced impairment of muscle function and coordination while female mice exhibit increased activity under TH excess

**Fig. 4:**
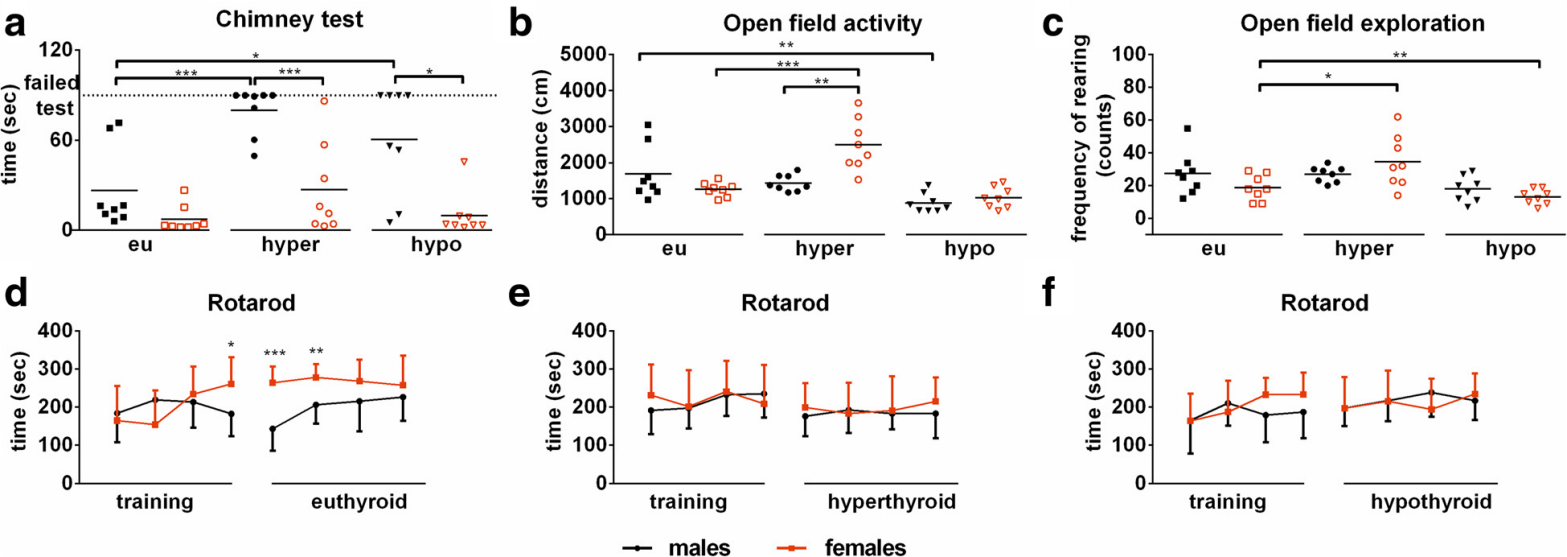
Behavioural assessment of male and female mice under T_4_ excess or deprivation. The chimney test was used to **a** examine muscle strength, tonus, and coordination of movements in male and female mice under euthyroid, hyperthyroid, or hypothyroid conditions. The open field was used to investigate activity and exploratory behaviour. **b** Total distance travelled was measured to assess activity, and **c** frequency of rearings was determined to assess exploratory behaviour. The rotarod test was used for an overall assessment of coordination and motor function in male and female mice before the start of treatment (training period) and under sham (**d**), T_4_ (**e**), or LoI/MMI/ClO_4_^−^ (**f**) treatment. Data are presented as mean ± SD, *N* = 8 animals/sex/treatment; two-way ANOVA followed by the Bonferroni post hoc analysis applied for sex and treatment effects of **a**–**c** and unpaired Student’s *t* test for sex effect of **d**–**f**, *p < 0.05, **p < 0.01, ***p < 0.001 represent multiple-testing or *t* test results

Muscle strength, tonus, and coordination of movements were examined by the chimney test. In general, female mice showed better performance in climbing up the tube than male mice (*m* ~26.57 vs *f* ~7.4 s, Fig. 4a). Hyper- and hypothyroidism resulted in decrease of muscle strength and coordination in female, but even more strikingly in male mice (*m* ~80.13 vs *f* ~27.11 s, *p* < 0.001, *F*_(1,28)_ = 23.94, *p* < 0.0001 for sex effect, *F*_(1,28)_ = 24.66, *p* < 0.0001 for treatment effect, and *F*_(1,28)_ = 5.266, *p* = 0.0294 for interaction (hyper) and *m* ~60.71 vs. *f* ~9.97 s, *p* < 0.05, *F*_(1,28)_ = 19.23, *p* = 0.0001 for sex effect, *F*_(1,28)_ = 5.3, *p* = 0.029 for treatment effect, and *F*_(1,28)_ = 3.923, *p* = 0.0575 for interaction (hypo)). Of note, performance in the chimney test was more impaired under TH excess than TH deprivation.

To investigate changes in activity and exploration behavior, an open field test was used. Overall activity was measured by total distance travelled, while exploration was quantified by the frequency of rearing events. Control animals showed no sex difference in open field parameters. Interestingly, T_4_ excess resulted in increased activity and exploratory behaviour only in female mice (*m* Δ ~ 257.9 cm vs *f* Δ ~ 1243.2 cm, *p* < 0.001 and *m* Δ ~ 8.6 vs f: Δ ~ 16.6 counts, *p* = 0.05), whereas LoI/MMI/ClO_4_^−^ treatment led to a decreased activity in male mice only (*m* Δ ~ 805.4 cm, vs *f* Δ ~ 227.2 cm, *p* < 0.01, Fig. 4b, c). Sex effect under hyperthyroidism was not significant (*F*_(1,28)_ = 3.164, *p* = 0.0861 for activity, *F*_(1,28)_ = 0.6493, *p* = 0.4272 for exploratory behaviour), but treatment effect reached statistical significance (*F*_(1,28)_ = 7.6, *p* = 0.0102 for activity and *F*_(1,28)_ = 10.60, *p* = 0.0030 for exploratory behaviour). Their interaction was significant for activity (*F*_(1,28)_ = 17.64, *p* = 0.0002), but not for exploratory behaviour (*F*_(1,28)_ = 1.064, *p* = 0.3112). While under hypothyroidism, no sex effect was found (*F*_(1,28)_ = 0.9943, *p* = 0.3272 for activity and *F*_(1,28)_ = 0.8467, *p* = 0.3654 for exploratory behaviour), treatment effect was significant (*F*_(1,28)_ = 12.86, *p* = 0.0013 for activity and *F*_(1,28)_ = 9.231, *p* = 0.0051 for exploratory behaviour). Furthermore, we found an interaction between sex and treatment on exploratory behaviour (*F*_(1,28)_ = 8.406, *p* = 0.0072), but not on activity (*F*_(1,28)_ = 4.033, *p* = 0.0544).

In contrast to these sex-specific modulations of TH impact on behaviour, no sex differences were noted for male and female mice on the rotarod test under eu-, hyper-, and hypothyroid conditions (Fig. 4d–f).

### Sex influences on serum thyroid function status in hyperthyroidism and liver function in hypothyroidism

**Fig. 5:**
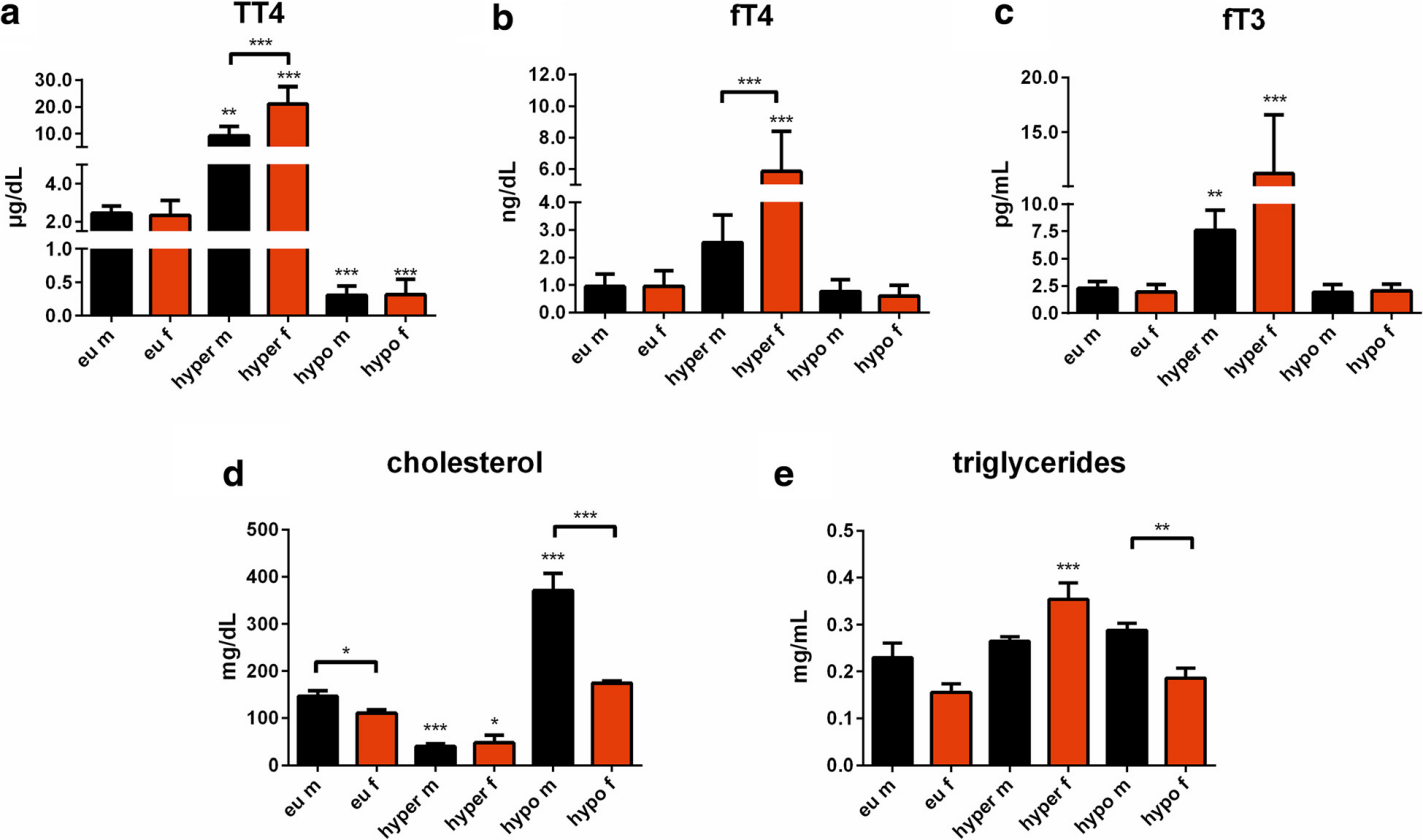
Serum TH status in euthyroid controls, T_4_ or LoI/MMI/ClO_4_^−^ treated male and female mice. **a** Total thyroxine (TT_4_), **b** free thyroxine (fT_4_), and **c** free triiodothyronine (fT_3_) concentrations were determined in sera by ELISA after 7 weeks of treatment. **d** Total cholesterol and **e** triglyceride serum concentrations were determined by ELISA at the end of experiment in sera of euthyroid, hyperthyroid, and hypothyroid mice of both sexes. Data are presented as mean ± SD, *N* = 8 animals/sex/treatment for TH concentrations, *N* = 4/sex/treatment animals for total cholesterol and triglyceride concentrations; two-way ANOVA followed by the Bonferroni post hoc analysis applied for sex and treatment effects, *p < 0.05, **p < 0.01, ***p < 0.001 above bars represent multiple-testing results

Serum TT_4_, fT_4_, and fT_3_ concentrations did not differ between euthyroid male and female mice (Fig. 5a–c). TSH serum concentrations of euthyroid male and female mice were 310 ± 170 mU/l and 290 ± 30 mU/l, respectively (±SEM, *n* = 4). T_4_ treatment resulted in marked sex differences in serum T_4_ and T_3_ status with 2.3-fold higher TT_4_ and fT_4_ concentrations in hyperthyroid females compared to male mice (Fig. 5a–c) and TSH concentrations below detection limit (<10 mU/l) in both sexes. LoI/MMI/ClO_4_^−^ treatment reduced TT_4_ concentrations below assay detection limit (<0.5 μg/dl) in both sexes (Fig. 5a–c) and increased TSH to 6830 ± 1070 mU/l and 7790 ± 1270 mU/l in male and female mice, respectively (±SEM, *n* = 4). Sex effects on TH serum parameters were observed for TT_4_ and fT_4_ under hyperthyroidism (TT_4_: *F*_(1,28)_ = 20.50, *p* = 0.0001; fT_4_: *F*_(1,28)_ = 10.80, *p* = 0.0027) but not for hypothyroidism (TT_4_: *F*_(1,28)_ = 0.09858, fT_4_: *p* = 0.7559; *F*_(1,28)_ = 0.2127, *p* = 0.6482) and not for fT_3_ (hyperthyroid: *F*_(1,28)_ = 2.485, *p* = 0.1261; hypothyroid_:_*F*_(1,28)_ = 0.1553, *p* = 0.6965). Treatment effects had an impact on TT_4_ concentrations (hyperthyroid: *F*_(1,28)_ = 95.74, *p* < 0.0001; hypothyroid_:_*F*_(1,28)_ = 165.8, *p* < 0.0001) and on fT_4_ and fT_3_ concentrations under hyperthyroidism (fT_4_: *F*_(1,28)_ = 41.32, *p* < 0.0001; fT_3:_*F*_(1,28)_ = 5.26, *p* < 0.0001). No treatment impact was observed under hypothyroidism for fT_4_ and fT_3_ (fT_4_: *F*_(1,28)_ = 2.316, *p* = 0.1393; fT_3_: *F*_(1,28)_ = 0.1645, *p* = 0.6882). Interaction of TH status and sex was found for TT4 and fT4 under hyperthyroidism (TT_4_: *F*_(1,28)_ = 21.26, *p* < 0.0001; fT_4_: *F*_(1,28)_ = 10.75, *p* = 0.0028) but not under hypothyroidism (TT_4_: *F*_(1,28)_ = 0.1272, *p* = 0.7241; fT_4_: *F*_(1,28)_ = 0.2350, *p* = 0.6316) and not for fT_3_ (hyperthyroid: *F*_(1,28)_ = 3.658, *p* = 0.0661; hypothyroid_:_*F*_(1,28)_ = 0.8460, *p* = 0.3656).

To examine the influence of sex on TH-dependent liver function, liver parameters were analyzed in sera collected at the end of treatment. While no changes were found in aspartate aminotransferase, creatine kinase, cholinesterase, and albumin serum concentrations (data not shown), a marked sex difference was found for total cholesterol (CHO) and triglyceride (TG) concentrations. Male mice exhibited higher CHO concentrations compared to female mice in the euthyroid state, and hypothyroidism led to significantly larger increases in serum CHO concentrations in male compared to female mice (*m* Δ ~ 224.6 mg/dl, *f* Δ ~ 63.9 mg/dl). In contrast, T_4_ treatment decreased total CHO concentrations in both sexes (*m* Δ ~ 106.9 mg/dl, *f* Δ ~ 63.1 mg/dl). Thus, sex difference in serum CHO levels disappeared during TH excess (*F*_(1,12)_ = 1.530, *p* = 0.2397), while deprivation led to an exaggeration (*F*_(1,12)_ = 35.44, *p* < 0.0001) (Fig. 5d). The treatment effect (*F*_(1,12)_ = 57.23, *p* < 0.0001 for hyperthyroidism and *F*_(1,12)_ = 54.66, *p* < 0.0001 for hypothyroidism) was considered significant and interacted with the sex effect (*F*_(1,12)_ = 3.799, *p* = 0.0075 for hyperthyroidism and *F*_(1,12)_ = 16.96, *p* = 0.0014 for hypothyroidism). Serum TG concentrations were not different in euthyroid males and females, but increased in hyperthyroid female mice only (*m* Δ ~ 0.035 mg/ml, *f* Δ ~ 0.198 mg/ml, *F*_(1,12)_ = 0.08541, *p* = 0.7751). However, a sex difference appeared by TH modulation and was exaggerated by LoI/MMI/ClO_4_^−^ treatment (*m* 0.288 mg/ml vs *f* 0.186 mg/ml, *p* < 0.05, *F*_(1,12)_ = 15.77, *p* = 0.0019) (Fig. 5e). The treatment effect was considered significant for hyperthyroidism (*F*_(1,12)_ = 20.98, *p* = 0.0006) and interacted with sex effect (*F*_(1,12)_ = 10.23, *p* = 0.0076), but not for hypothyroidism (*F*_(1,12)_ = 3.983, *p* = 0.0692; *F*_(1,12)_ = 0.3958, *p* = 0.5410 for interaction).

### Evidence for a distinct impact of sex on cellular TH effects on gene expression in target organs brown adipose tissue, heart, and liver

**Fig. 6:**
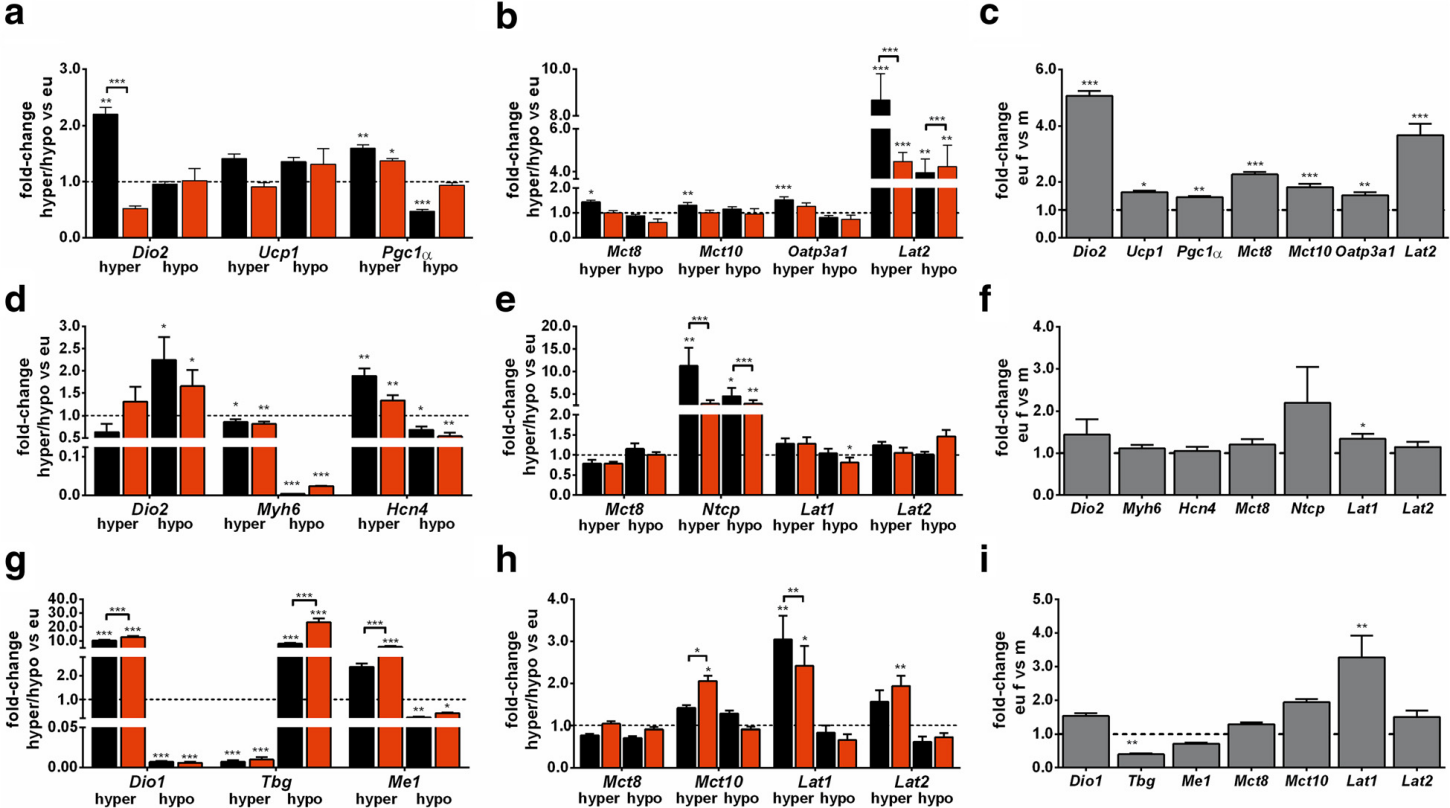
TH effects in brown adipose tissue (BAT), heart, and liver of male and female mice. Fold changes of representative TH-responsive genes were measured by quantitative RT-PCR in **a** BAT, **d** heart, and **g** liver tissue of hyperthyroid or hypothyroid mice of both sexes and normalized to tissue samples of euthyroid control mice. For BAT *Dio2*, *Ucp1*, and *PGC1α* expression; for heart *Dio2*, *Myh6*, and *Hcn4* expression; and for liver *Dio1*, *Tbg*, and *Me1* expression were quantified. Additionally, mRNA expression of TH transporter genes were analyzed in **b** BAT, **e** heart, and **h** liver. For BAT: *Mct8*, *Mct10*, *Oatp3a1*, *Lat2*, for heart: *Mct8*, *Ntcp*, *Lat1*, *Lat2*, and for liver: *Mct8*, *Mct10*, *Lat1*, *Lat2*. Furthermore, euthyroid sex comparison was analyzed in **c** BAT, **f** heart, and **i** liver of all genes, and gene expression in female tissues was normalized to male samples. Data are presented as mean ± SD, *N* = 5–7 animals/sex/treatment; unpaired Student’s *t* test, *p < 0.05, **p < 0.01, ***p < 0.001 represent *t* test results

Expression of TH-responsive genes and TH transporters was studied by quantitative RT-PCR in BAT, heart, and liver of male and female mice under T_4_ excess, TH deprivation, and euthyroid conditions (Fig. 6a–i). A distinct and organ-specific pattern of sex variation in gene expression was observed. In brown adipose tissue, marked sex-specific alterations in *Dio2* transcript levels were detected in hyperthyroid (upregulation in male, downregulation in female mice) and for *Lat2* in hyper- and hypothyroid animals (Fig. 6a, b). Additionally, sex-dependent variation was found for expression of all investigated target genes and TH transporters in euthyroid mice (Fig. 6c). In contrast to this data, very little or no sex impact was found on target gene or TH transporter gene expression in heart neither in euthyroid controls nor in response to T_4_ or LoI/MMI/ClO_4_^−^ treatment (Fig. 6d–f). In fact, for most investigated genes, a distinctly higher expression was found in heart tissue of male mice irrespective of thyroid function status. In line with the contribution of liver and BAT to metabolic features of thyroid dysfunction, significant sex-specific alterations for, e.g., *Dio1*, *Tbg*, and *Me1* as well as *Mct10* and *Lat1* expression were obvious with the manipulation of thyroid status (Fig. 6g–h), while livers of male and female euthyroid control mice showed little sex variation in target gene and TH transporter expression (Fig. 6i).

## Discussion

The female preponderance of thyroid dysfunction is well known; however, whether gender has an impact on manifestation and outcome of hyper- and hypothyroidism in men and women has not been well studied, and the molecular mechanisms are still unclear. Using a murine model, we found marked sex differences in functional behaviour and metabolic and biochemical parameters in hyper- and hypothyroid mice. Sex of mice did not alter the general response of body weight and heart rate to the manipulation of thyroid status; only the extent of these changes is somewhat different. In addition, a distinct sex variation in cellular TH effects on gene expression was observed in BAT, heart, and liver on the basis of TH target and TH transporter gene expression analysis.

### Sex difference for water intake reverses during TH deprivation

A clear sex difference could be observed for water intake in euthyroid and hyperthyroid conditions as females consumed more water than male mice (Fig. 2g, h). Strikingly, this is reversed in hypothyroidism and female mice consume significantly less water than their male counterparts (Fig. 2i). This observation could indicate that female mice taste bitter substances differently due to altered taste receptor distributions. However, studies of taste receptors in mice either addressed only male mice [18] or observed no sex differences [19]. Preferences for the primary taste qualities sweet, sour, bitter, salty, and umami showed no sex differences for C57BL/6 mice [20]. Thus, the supplemented drugs might be recognized not by usual taste receptors but different receptor types in female and male mice. However, decreased water consumption did not affect the success of LoI/MMI/ClO_4_^−^ treatment as TT_4_ serum concentrations were below limit of detection and serum TSH was highly elevated to comparable values for both sexes. The manipulation of TH status might also affect hormonal regulation of fluid intake, water and salt homeostasis, an observation compatible with altered water metabolism in hypothyroid patients [21, 22] and increased hypothalamic vasoactive intestinal polypeptide-immunoreactive neuron expression in hypothyroid rats [23]. For other phenotypic traits related to the manipulation of TH concentration, sex-specific differences persisted (e.g., body temperature in hyper- and hypothyroidism), were exaggerated (e.g., food intake in hyperthyroidism) or disappeared (e.g., BW change in hypothyroidism).

### TH excess results in distinct exaggeration of behavioural traits in male vs female mice

Behavioural assessment of our mice consisted of measurements of locomotor activity, motor function, and coordination tests. TH manipulation resulted in increased activity and exploratory behaviour during TH excess in female mice only, while decreased activity under TH deprivation was present in male mice only (Fig 4b, c). Previous studies with adult-onset hypothyroidism in male mice support our observation of decreased locomotor activity, with no consistent findings for anxiety [24, 25]. Impact of sex and TH excess on locomotor activity has not been addressed so far. For euthyroidism, a higher locomotor activity as well as increased voluntary exercise of females compared to male mice has previously been described [26, 27] and was confirmed in our study. The chimney test investigates motor coordination and muscular strength and showed higher impairment of performance by T_4_ administration than by LoI/MMI/ClO_4_^−^ treatment for both sexes (Fig. 4a). Male mice showed more pronounced affliction of chimney test performance under the manipulation of TH status. Since the rotarod test which evaluates motor coordination and balance in movements showed no sex or treatment differences (Fig. 4d–f), we suspect that altered muscle function could be responsible for the observed effects in the chimney test. To our knowledge, our study is the first to assess muscle strength under hyper- and hypothyroid conditions for mice of both sexes. When assessing behaviour, it is important to distinguish whether the differences are due to a compromised muscle function or neurological impairment. Comparison of mice of both sexes showed generally shorter contraction of isolated muscles in male mice [28]. Therefore, it has been suggested that male muscles are generally faster and have a higher maximum power output than female muscles. On the other hand, female muscles are generally more fatigue resistant, recover faster, and show less mechanical damage after exercise [28, 29]. As central nervous system and peripheral nervous system coordinate muscle function, it is not unreasonable to conclude that both neurological and primary muscular factors might contribute to observed sex differences [30, 31]. These aspects remain to be investigated.

### Sex difference in body temperature persists throughout thyroid dysfunction and is reflected by cellular TH effects on gene expression in BAT

Persisting sex differences in eu- and hyperthyroidism between male and female mice were found for body temperature with higher values in female mice (Fig. 3a). It has previously been described that euthyroid female mice have higher body temperature than male mice, but this is not explained by classical TH serum concentrations in euthyroid or hyperthyroid animals [26, 32]. As BAT is a TH target organ and involved in the regulation of body temperature [33], gene expression analysis was performed for TH target genes and TH transporter expression. With the manipulation of TH status, TH-responsive sex difference in gene expression was only found for *Dio2*, which increased in BAT of hyperthyroid male mice but decreased in BAT of hyperthyroid female mice (Fig. 6a). However, we cannot extrapolate from transcript concentration on enzyme activity, as previous studies showed different regulation of *Dio2* under TH influence [34]. No sex difference was found for the investigated TH-responsive genes under TH deprivation. This supports the observed persisting sex differences in body temperature, as TH manipulation resulted in little sex differences of TH-responsive gene expression in BAT. For TH transporter genes, only *Lat2* was higher expressed in BAT of hyperthyroid male compared to female mice, however, *Lat2* gene expression was also increased in BAT of hypothyroid female compared to male mice (Fig. 6b). *Lat2* is known to transport not only TH (preferentially diiodothyronines) but also small neutral amino acids [35, 36]. Thus, it might reflect sex-different needs for amino acid transport in BAT during TH dysfunction.

For interpretation of cellular TH effects on gene expression in BAT, it is important to remember that two mechanisms play a role in BAT activation and thermogenesis. Firstly, adjustment of body temperature in response to thermal stress which occurs due to housing temperature (23 °C instead of thermoneutrality at 30 °C) and therefore chronically augmented metabolism [37]. Secondly, activation of basal energy expenditure by TH results in increased thermogenesis [38, 39]. The balance between these two mechanisms has been addressed in recent studies under TH deprivation and showed that BAT thermogenic program was only down-regulated when hypothyroidism was combined with thermoneutrality [40]. In our study, mice were not kept under thermoneutral condition; hence, we expect an overlap between adjustment to thermal stress, and activation or inactivation of basal energy expenditure by TH excess or deprivation, respectively.

### Sex difference in heart rate disappears with hyper- and hypothyroidism

Euthyroid female mice showed higher HR compared to males, but this sex difference disappeared with a hyper- or hypothyroid state (Fig. 3b). We asked whether these changes in sex impact would also be mirrored by cellular TH effects and hence performed gene expression analysis in heart tissue. No sex difference in the expression of investigated TH-responsive genes was found. Similarly, for TH transporters, only *Ntcp* showed different expression in male and female heart tissues irrespective of TH dysfunction (Fig. 6e). Hence, this data does not explain why sex difference in HR disappears with the manipulation of TH status, and it is therefore likely that the investigated genes do not reflect the total organ response. Sex-specific impact on neuronal regulation of heart rate and function might lead to these changes independent from myocardial gene expression [41].

### TH-dependent liver function and metabolism is sex-specific

It is well known that TH influences serum total CHO concentrations [42–46]. A correlation with serum TH status was confirmed by changes in CHO serum concentrations of our mice. A sex difference in CHO levels was augmented during LoI/MMI/ClO_4_^−^ treatment and disappeared under T_4_ administration (Fig. 5d). Since male mice showed more pronounced changes than female mice, we suggest that CHO metabolism in livers of male mice may be more sensitive to TH manipulation compared to female mice. This observation is supported by the known sex-dependent regulation of *Cyp7a1* in transgenic mice, where hyperthyroidism decreased *Cyp7a1* in male mice, while no regulation could be found in female mice [43]. In addition, studies on changes in other functional parameters in liver tissues revealed sex-dependent regulation of tight junction proteins, such as claudin-1 and claudin-2 in cholangiocytes and hepatocytes of male and female animals under hypothyroid conditions. Further experiments including analysis of CHO synthesis pathways led to the hypothesis that claudins may be relevant for reversal of sex hormone-related susceptibility to gallstone formation in hypothyroidism [6]. On the other hand, TG serum concentration increased in hyperthyroid female mice only (Fig. 5e) and suggest a different TG metabolism response to TH in male and female mice. Gene expression profile shows more pronounced TH-dependent regulation in the liver of female compared to male mice. Thus, a higher expression of TH-responsive genes *Dio1* and *Me1* was noted in hyperthyroidism in female mice liver, and *Tbg* transcripts were elevated in hypothyroid female liver compared to male mice. For TH transporters, *Mct10* gene expression was increased in hyperthyroid female mice and for *Lat1*, a higher expression was observed in hyperthyroid male mice (Fig. 6g, h).

### Possible influence of estrogens on TT_4_ and fT_4_ serum concentrations during TH excess

One very obvious sex-specific alteration was the increase in TH serum concentrations in female hyperthyroid mice. In fact, 2.3-fold higher TT_4_ and fT_4_ serum concentrations were measured in female mice after 7 weeks of T_4_ treatment (Fig. 5a–c). This is in agreement with previous findings showing a 2-fold increase in TT_3_ serum concentrations in female mice under T_3_ treatment, whereas male mice revealed only 50 % increase in TT_3_ serum concentrations [47]. In contrast, under TH deprivation, no sex differences in serum TT_4_, fT_4_, fT_3_, and TSH concentrations were found in our mice. Sex differences in thyroid function parameters, such as TSH and classical thyroid hormones T_4_ and T_3_, have also been addressed in epidemiological studies; however, no consistent findings were reported [48–50]. Other studies using murine models have described sex differences in thyroid function parameters at least for some strains and indicate that serum TH status needs to be considered when studying TH action [7, 51]. Thus, for every genotype, TH serum concentrations should be measured in a sex-matched manner.

When studying sex differences in animal models or humans, it is likely that the most elaborated differences between male and female individuals are related to gonadal hormones. The connection between TH and estrogens has been a subject of extensive research. One major effect is an increase in thyroxine-binding globulin capacity by estrogens [52, 53]. In our mice study, we observed increased TT_4_ serum concentration in hyperthyroid female compared to male mice, which could be explained by higher binding capacity of thyroxine-binding globulin. However, this does not explain the sex-dependent difference in fT_4_ concentrations, which were also significantly higher in female compared to male mice. Secondly, interactions between gonadal and thyroid hormones occur through the hypothalamic-pituitary-thyroid and the hypothalamic-pituitary-gonadal axis; thus, hyper- and hypothyroidism can interfere with the gonadal axis resulting in infertility in particular in the female organism. Finally, there was no apparent direct linear correlation between phenotype and classical T_4_/T_3_ ratio in relation to observed sex variation in phenotypical traits. Thus, although higher TT_4_ and fT_4_ serum concentrations were present in hyperthyroid female mice, they show exaggeration only in motor activity and exploration while other traits of hyperthyroidism are less prominent than in male animals.

## Conclusions

We showed that sex is an important modifier of TH action resulting in distinct phenotypic, metabolic, and biochemical traits of hyper- and hypothyroidism in male and female mice. Importantly, a direct linear link between TH serum concentrations and sex-dependent phenotype was not observed. The wide spectrum of key players in TH action, such as distinct circulating TH derivatives and metabolites, tissue-specific TH transporters, non-genomic TH effects, and classical nuclear TH action, illustrates the complex situation in an intact organism [54]. It seems crucial to address possible sex difference under natural conditions, and further analysis of sex-specific traits will require additional studies using, e.g., gonadectomized animals.

## Additional file

Additional file 1: Table S1. Oligonucleotides used for amplification of house-keeping genes, TH responsive genes, and TH transporters by real-time PCR. (DOCX 28 kb)

## Abbreviations

BAT: Brown adipose tissue
BW: Body weight
CHO: Cholesterol
ClO_4-_: Perchlorate
ECG: Electrocardiography
f: Female
fT_3_: Free 3,3′,5-triiodothyronine
fT_4_: Free thyroxine
HR: Heart rate
LoI: Low-iodine diet
m: Male
MMI: Methimazole
qRT-PCR: Quantitative real-time PCR
T_3_: 3,3′,5-triiodothyronine
T_4_: Thyroxine
TG: Triglycerides
TH: Thyroid hormone
TSH: Thyroid-stimulating hormone
TT_4_: Total thyroxine
