# Supplementary material for: Sex-specific phenotypes of hyperthyroidism and hypothyroidism in mice
Source: Biol Sex Differ. 2016 Aug 24;7(1):36. doi: 10.1186/s13293-016-0089-3 (PMC4995626; doi:10.1186/s13293-016-0089-3)
Supplement: Additional file 1: Table S1. — Oligonucleotides used for amplification of house-keeping genes, TH responsive genes, and TH transporters by real-time PCR. (DOCX 28 kb) [file 13293_2016_89_MOESM1_ESM.docx]

**Suppl. Table 1** Oligonucleotides used for amplification of house-keeping genes, TH responsive genes and TH transporters by real-time PCR.

| gene | Forward primer | Reverse primer |
| --- | --- | --- |
| *18S* | CGGCTACCACATCCAAGGAA | GCTGGAATTACCGCGGCT |
| *Ppia* | CTTGGGCCGCGTCTCCTTCG | GCGTGTAAAGTCACCACCCTGGC |
| *RPL 13a* | GGGCAGGTTCTGGTATTGGA | GGGGTTGGTATTCAATCCGCT |
| *Gapdh* | CCTCGTCCCGTAGACAAAATG | TGAAGGGGTCGTTGATGGC |
| *ß-Actin* | CTGTCGAGTCGCGTCCA | TCATCCATGGCGAACTGGTG |
| *Polr2a* | CTTTGAGGAAACGGTGGATGTC | TCCCTTCATCGGGTCACTCT |
| *Dio1* | GGGCAGGATCTGCTACAAGG | CGTGTCTAGGTGGAGTGCAA |
| *Dio2* | GTGACTGGGGAAGCAGAGTG | AGTTTAACCTGTTTGTAGGCATC |
| *Tbg* | TGGGCATGTGCTATCATCTTCA | GAGTGGCATTTTGTTGGGGC |
| *Me1* | TAAGGGTCGTGCATCTCTCAC | TGCAGCAACTCCTATGAGGG |
| *Myh6* | CAGACAGAGATTTCTCCAACCCA | GCCTCTAGGCGTTCCTTCTC |
| *Hcn4* | CAGCGTCAGAGCGGATACTT | CTTCTTGCCTATGCGGTCCA |
| *Ucp1* | GGATGGTGAACCCGACAACT | CTTGGATCTGAAGGCGGACT |
| *PGC1α* | TCTCAGTAAGGGGCTGGTTG | AGCAGCACACTCTATGTCACTC |
| *Mct8* | CTCCTTCACCAGCTCCCTAAG | ACTTCCAGCAGATACCACACC |
| *Mct10* | CGTGAGTGTCTTCACGGACA | CGATGGAGCTTACAAAAGAACTGG |
| *Lat1* | AGCGTCCCATCAAGGTGAAT | GGGCTTGTTCTTCCACCAGA |
| *Lat2* | AACAACACCGCGAAGAACCA | GGAGCCAATGATGTTCCCTACAA |
| *Oatp3a1* | CGCTACGGAAACAACTCAGC | GAGTCCGTCTGGCATTCACA |
| *Ntcp* | GGGGACATGAACCTCAGCATT | CCCTATGGCGCAAGGAATGA |
